# Comparison of oral microbiota in tumor and non-tumor tissues of patients with oral squamous cell carcinoma

**DOI:** 10.1186/1471-2180-12-144

**Published:** 2012-07-20

**Authors:** Smruti Pushalkar, Xiaojie Ji, Yihong Li, Cherry Estilo, Ramanathan Yegnanarayana, Bhuvanesh Singh, Xin Li, Deepak Saxena

**Affiliations:** 1 Department of Basic Sciences and Craniofacial Biology, New York University College of Dentistry, 345 E, 24th Street, Room 921B, New York, NY, 10010, USA; 2 Department of Chemical and Biological Sciences, Polytechnic Institute of NYU, New York, NY, USA; 3 Dental Service, Department of Surgery, Memorial Sloan-Kettering Cancer Center, New York, NY, USA; 4 Laboratory of Epithelial Cancer Biology, Memorial Sloan-Kettering Cancer Center, New York, NY, USA

**Keywords:** Microbial diversity, Oral squamous cell carcinoma, Microbiota, Tumor, Non-tumor

## Abstract

**Background:**

Bacterial infections have been linked to malignancies due to their ability to induce chronic inflammation. We investigated the association of oral bacteria in oral squamous cell carcinoma (OSCC/tumor) tissues and compared with adjacent non-tumor mucosa sampled 5 cm distant from the same patient (*n = 10*). By using culture-independent 16S rRNA approaches, denaturing gradient gel electrophoresis (DGGE) and cloning and sequencing, we assessed the total bacterial diversity in these clinical samples.

**Results:**

DGGE fingerprints showed variations in the band intensity profiles within non-tumor and tumor tissues of the same patient and among the two groups. The clonal analysis indicated that from a total of 1200 sequences characterized, 80 bacterial species/phylotypes were detected representing six phyla, *Firmicutes*, *Bacteroidetes*, *Proteobacteria*, *Fusobacteria*, *Actinobacteria* and uncultivated *TM7* in non-tumor and tumor libraries. In combined library, 12 classes, 16 order, 26 families and 40 genera were observed. Bacterial species, *Streptococcus sp*. oral taxon 058, *Peptostreptococcus stomatis*, *Streptococcus salivarius*, *Streptococcus gordonii*, *Gemella haemolysans, Gemella morbillorum*, *Johnsonella ignava* and *Streptococcus parasanguinis I* were highly associated with tumor site where as *Granulicatella adiacens* was prevalent at non-tumor site. *Streptococcus intermedius* was present in 70% of both non-tumor and tumor sites.

**Conclusions:**

The underlying changes in the bacterial diversity in the oral mucosal tissues from non-tumor and tumor sites of OSCC subjects indicated a shift in bacterial colonization. These most prevalent or unique bacterial species/phylotypes present in tumor tissues may be associated with OSCC and needs to be further investigated with a larger sample size.

## Background

Oral cancer is one of the ten most prevalent cancers in the world with more than 90% of mouth neoplasms being squamous cell carcinoma that has its origin from the oral mucosa [1-3]. During the year 2011, in United States, approximately, 39,400 new cases and 7,900 deaths were estimated attributing to cancer of oral cavity and pharynx [4]. Five year survival rates for persons with this medical condition are currently only 60.9% [4]. The early detection of oral cancer at initial stages is critical and requires less radical treatment for patient’s survival and improving quality of life. The pathogenesis of OSCC is attributed mainly to smoking, heavy alcohol consumption and smokeless tobacco products [5-7]. Other possible risk factors include viral infections [8,9], infection with *Candida* species [10], periodontitis [11,12], poor oral hygiene [13], poor dental status [14] and chronic bacterial infections and inflammation [5,6,15-17].

The association of bacterial infection and cancer is classically represented by *Helicobacter pylori* and its involvement in gastric adenocarcinoma and mucosa associated lymphoid tissue (MALT) lymphoma [18]. Some studies suggests possible link between *Salmonella typhi* and gall bladder cancer, *Streptococcus bovis* and colon cancer, *Chlamydophila pneumoniae* and lung cancer, *Bartonella* species and vascular tumor formation, *Propionibacterium acnes* and prostate cancer and *Escherichia coli* in inflammatory bowel disease with increased risk of colon cancer [15,19,20]. These findings were confirmed by using several animal (mice) models for *Helicobacter hepaticus* associated with hepatocellular carcinoma [21], colon cancer [22] and cancer in mammary glands [23]. There is growing evidence that bacterial infection is causally related to carcinogenesis.

Several mechanisms for possible bacterial association in carcinogenesis may include chronic infection by evasion of immune system and immune suppression [24], or induction of chronic inflammation [25], or direct or indirect interference with eukaryotic cell cycle and signaling pathways [8,15], or via metabolism of potential carcinogenic substances [7]. The host cells are susceptible to microbial endotoxins (lipopolysaccharides), enzymes (proteases, collagenases, fibrinolysin and phospholipase) and their metabolic by-products (hydrogen sulfide, ammonia and fatty acids) and may directly induce mutations in tumor suppressor genes and proto-oncogenes or alter signaling pathways that affect cell proliferation and/or survival of epithelial cells [8,15,24]. Microorganisms and their products activate neutrophils, macrophages, monocytes, lymphocytes, fibroblasts and epithelial cells to generate reactive species (hydrogen peroxide and oxygen radicals), reactive nitrogen species (nitric oxides), reactive lipids and metabolites (malondialdehyde and 4-hydroxy-2-nonenal) and matrix metalloproteases. These compounds can induce DNA damage in epithelial cells [20] and directly affect tumor growth by activating tumor cell toll-like receptors (TLR) that eventually leads to nuclear translocation of the transcription factor NF-kB and cytokines production [26,27]. These cytokines are produced in dysregulated fashion and have roles in cell growth, invasion and interruption of tumor suppression, immune status and even survival [28]. It is unclear whether these mediators are critical for the development and/or growth of tumors and/or whether they constitute a permissive environment for the progression of malignancies [29]. Elevated levels of certain proinflammatory, proangiogenic NF-kB dependent cytokines TNF-α, IL-1, IL-6, IL-8, GM-CSF and VEGF were observed in serum, saliva, and tissue specimens of patients with oral cancer [30,31].

The oral cavity harbors diversified microflora with more than 750 distinct bacterial taxa [14] that colonize host tissues and co-aggregate with one another [32]. Any loss in integrity of oral epithelial barrier exposes the underlying tissues to various aerobic and anaerobic microflora of oral cavity [33]. Hence, the local and systemic polymicrobial mucosal infections may be a result of invading potentially pathogenic microorganism of extra-oral origin or a shift within the normal commensal microflora taken up by opportunistic microflora in immuno-compromised individuals [33].

Previous studies on oral microbiota of patients with and without OSCC using culture-dependent [10,33-36] and culture-independent [37-40] techniques indicated bacterial community profiles to be highly correlated at phylum level but diverse at genus level. Hooper et al. [34,38] observed that most of the taxa in non-tumor and tumor tissues were known members of oral cavity and majority of those in tumor tissue were saccharolytic and aciduric species. Our studies on bacterial diversity in saliva samples by 454 pyrosequencing revealed 244 bacterial OTUs exclusive to OSCC patients (*n = 3*) as compared to non-OSCC controls (*n = 2*) [40]. To establish the role of bacteria in OSCC, it is important to determine the differences in the colonization of oral bacteria in non-tumor and tumor tissues. We hypothesized that any differences in bacterial profile at tumor sites in contrast to non-tumor sites may indicate its involvement in tumor pathogenesis.

We used 16S rRNA based two culture-independent methods, denaturing gradient gel electrophoresis and sequencing to elucidate the total oral microbiota in non-tumor and tumor tissues of OSCC patients. This may facilitate to identify the microbial transition in non-tumor and tumor tissues and understand better the association of bacterial colonization in OSCC.

## Methods

### Subject selection and sampling procedure

Twenty oral tissue samples, 10 each from non-tumor and tumor sites of 10 patients with squamous cell carcinoma of oral tongue and floor of the mouth, median age 59 years (53% male and 47% female) were obtained from Memorial Sloan-Kettering Cancer Center (MSKCC) Tissue Bank, refer Estilo et al. and Singh et al. [41-43] for clinical details. The subjects had a history of smoking and drinking and were not on antibiotics for a month before sampling. The study was approved by institutional review boards at MSKCC and NYU School of Medicine and written informed consent was obtained from all participants involved in this study. The tissues were collected following guidelines established by Institutional Review Board at MSKCC and tumors were identified according to tumor-node-metastasis classification by American Joint Committee on Cancer/Union International Cancer Center.

For this study, to have a homogenous sample population and to control the effect of confounding factors on bacterial colonization, we used non-tumor tissue from upper aerodigestive tract as a control, resected 5 cm distant from the tumor area or contralateral side of the same OSCC patient and confirmed histologically as normal mucosae [42]. The tissue samples were processed to include all bacteria (on the surface and within the tissue) to detect the total bacterial diversity in oral mucosa. The samples were procured and stored at −80°C till further analysis.

### DNA extraction from tissue samples

Tissue specimens were pretreated as mentioned earlier by Ji et al. [44]. Briefly, the tissues were suspended in 500 μL of sterile phosphate-buffered saline (PBS), vortexed for 30 seconds and sonicated for 5 and 10 seconds respectively. Proteinase K (2.5 μg/mL) was added for digestion and incubated overnight at 55°C, if required, homogenized with sterile disposable pestle and vortexed. The bacterial genomic DNA was extracted by modified Epicentre protocol (Epicentre Biotechnologies, Madison, WI) and purified with phenol-chloroform extraction [45]. Samples were analyzed qualitatively and quantitatively by NanoDrop ND 1000 spectrophotometer (NanoDrop Technologies Inc., Wilmington, DE). All samples were stored at −20°C till further analysis. For PCR assays, the DNA concentration was adjusted to 20 ng/μL.

### 16S rDNA amplification

The 16S rDNA samples were amplified as described earlier [44] using universal primer pair 8F and 1492R [46-48] for cloning. PCR reactions were run at 95°C for 5 min, followed by 30 cycles of denaturation at 95°C for 1 min, annealing at 52°C for 1 min, and elongation at 72°C for 1 min with final elongation at 72°C for 5 min. The nested PCR was performed targeting V4-V5 hypervariable region with another set of eubacterial primers, prbac1 and prbac2 [49] with 40-nucleotide GC clamp [50] added to 5’ end of prbac1 for DGGE assay. The conditions of nested PCR were 3 min preheating at 94°C, 35 cycles each at 94°C (30 seconds), 63°C (40 seconds), and 72°C (1 min), final extension at 72°C for 7 min. For both PCR assays, the reaction system was 50 μL comprising 1 μL DNA template, 5 U Taq DNA polymerase (Invitrogen, Carlsbad, CA), 5 μL 10x PCR buffer, 1.5 μL MgCl_2_ (50 mM), 4 μL dNTP mixture (2.5 mM each) and 50 pmol of each primer.

### DGGE assay

PCR products from nested PCR were analyzed for sequence polymorphism on 40% to 60% linear DNA denaturing gradient polyacrylamide gel, 8.0% w/v. 30 μL of each were loaded on DGGE gel with standard species-specific DGGE reference markers [40,51] resolved by DCode system (Bio-Rad, Hercules, CA). The gels were run for 16 hr at 58°C and 60 V in 1x Tris-acetate-EDTA (TAE) buffer, pH 8.5 and stained with ethidium bromide solution (0.5 μg/mL) for 15 min. The images were digitally documented using Alpha Imager 3300 system (Alpha Innotech Corporation, San Leandro, CA).

### Cluster and statistical analyses of DGGE microbial profiles

DGGE gel pattern of amplicons were analyzed with the aid of Fingerprinting II Informatix Software (Bio-Rad) and interpreted statistically [52]. The gels were normalized with DGGE standard markers and background subtracted using mathematical algorithms based on spectral analysis of overall densitometric curves. The similarity among samples was calculated by Dice coefficient. Dendrogram was configured from average matrix by Ward analysis. The variations in microbial profiles of non-tumor and tumor tissues were assessed by comparing inter- and intra- groups DGGE profiles of PCR amplified segments. Differences were examined for statistical significance using Mann–Whitney *U* test and Chi-square test. Statistical analysis was performed using SPSS software v. 17.0 (SPSS inc., Chicago, IL).

### Cloning and sequencing

PCR amplicons were ligated to pCR4-TOPO vector and transformed into *E. coli* TOP10 cells using TOPO-TA cloning kit according to manufacturer’s instructions (Invitrogen). From each sample, about 95–96 clones were picked and a total of 1914 clones were sequenced unidirectional (Beckman Coulter Genomics, Beverly, MA) using BigDye Terminator v3.1 and 806r sequencing primer and analyzed on ABI PRISM 3730*xl* coupled with Agencourt CleanSEQ dye terminator removal for generation of long high quality Sanger sequencing reads. About 1200 sequences with Phred 20 and average read length of 700 bases were trimmed by removing vector sequences and adjusted for quality values. The majority of single sequence read length was between 350–900 bases. All the trimmed sequences were verified manually for vector sequences using EMBOSS pairwise alignment algorithms [53].

### Phylogenetic analysis of sequences in group specific libraries

Sequences were aligned with Greengenes Nast aligner ( http://greengenes.lbl.gov) [54] and then checked for chimeras on greengenes chimera check program supported by Bellerophon [54,55]. About 0.7% sequences were chimeric and eliminated from analysis. The sequences with 350 to 900 bases were analyzed against 16S rRNA reference sequences of Human Oral Microbiome Database (HOMD, version 10.1) [56,57]. Sequence identification requires a single read of approximately 350 to 500 bases [58]. The threshold assigned for BLAST identification of partial sequences was ≥98% similarity for species/phylotypes. Majority of sequences could be identified to species/phylotype level. The sequences with <98% identity were characterized only till genus level and considered unclassified sequences at species level. Non-tumor and tumor libraries were constructed from clonal analysis. These sequences were also analyzed using Ribosomal Database Project (RDP, Release 10) [59]. The relative distribution of abundance for phylogenetic groups in two different libraries was compared by chi-square test. The intra- (within) and inter- (between) groups bacterial species/phylotypes in 16S clonal libraries were evaluated. In analysis, for representation of bacterial taxa, the term, *specie*s refers to named cultivated species and unnamed cultivated taxon and *phylotypes* refers to non-cultivable or yet- uncultured species.

### Diversity and richness estimation of group specific libraries

Richness estimator, Chao1 was determined by ESTIMATES v. 7 [60] and rarefaction curves, rank abundance and diversity indices performed in PAST v. 1.89 [61]. The species rarefaction of the entire dataset was computed by individual rarefaction method. The percentage of coverage was calculated by Good’s method using equation (1−n/N) x 100, where n is number of singletons represented by one clone in the library and N is total number of sequences in the sample library [62]. The diversity of each sampled sequence set was estimated by using Shannon (*H’*) and Simpson (1–*D*) indices within PAST application. The Shannon index of evenness was calculated with the formula *E* = e^H/S, where H is Shannon diversity index and S is number of taxa (species/phylotypes) in that group.

## Results

**Figure 1:**
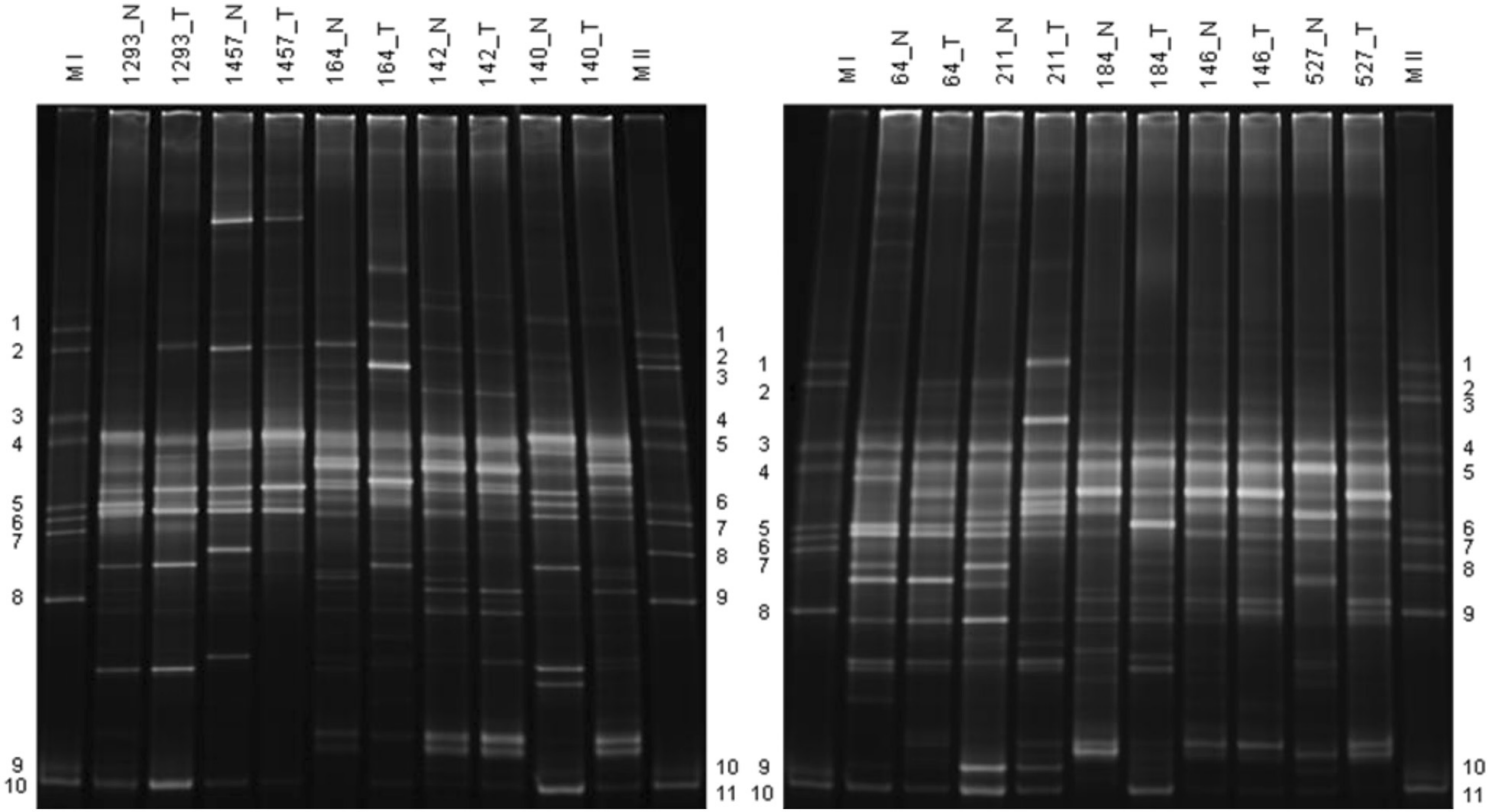
**DGGE profile of microbial communities from two clinically distinct non-tumor and tumor groups.** N–Non-tumor; T–Tumor; Marker I & II: DGGE reference markers correspond to 16S rRNA gene fragments from quoted specific bacterial species [Marker I: 1. *Fusobacterium nucleatum* subsp. *vincenti* (ATCC 49256); 2. *Fusobacterium nucleatum* subsp. *nucleatum* (ATCC 25586); 3. *Streptococcus sanguinis* (ATCC 10556); 4. *Streptococcus oralis* (ATCC 35037); 5. *Streptococcus salivarius* (ATCC 7073); 6. *Streptococcus mutans* (UA 159); 7. *Lactobacillus paracasei* (ATCC 25598); 8. *Porphyromonas gingivalis* (ATCC 33277); 9. *Actinomyces odontolyticus* (ATCC 17929);10. *Actinomyces naeslundii* (ATCC 12104), Marker II: 1. *F. nucleatum* subsp. *vincenti* (ATCC 49256); 2. *F. nucleatum* subsp. *nucleatum* (ATCC 25586); 3. *Bacteroides forsythus* (ATCC 43037); 4. *S. sanguinis* (ATCC 10556); 5. *S. oralis* (ATCC 35037); 6. *Veillonella parvula* (ATCC 17745); 7. *Prevotella intermedia* (ATCC 25611); 8. *Aggregatibacter actinomycemcomitans* (ATCC 43717); 9. *P. gingivalis* (ATCC 33277); 10. *A.odontolyticus* (ATCC 17929); 11. *A. naeslundii* (ATCC 12104)].

**Figure 2:**
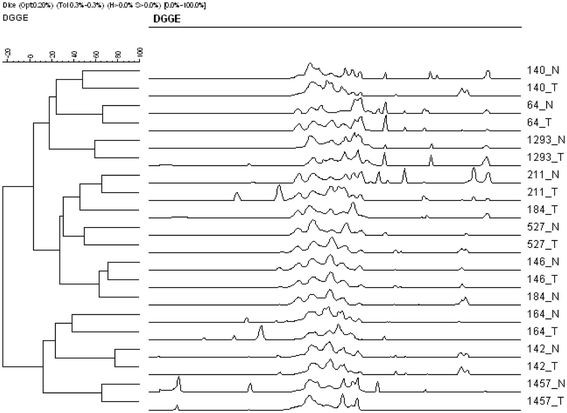
**Dendrogram representing the fingerprinting intensity profile of two clinically distinct samples from non-tumor and tumor tissues.** N–Non-tumor; T–Tumor.

In this study, DGGE was used as a method for preliminary and rapid assessment of bacterial diversity in tumor and non-tumor tissues. DGGE gel profiles of non-tumor and tumor samples (n = 20) were analyzed after normalization of gels with species-specific markers (Figure 1). In total, 68 and 64 bands were distinct to non-tumor and tumor groups respectively of which 8 bands were exclusive to non-tumor samples while 4 bands exclusive to tumor group. Each band may correspond to one or more bacterial species. The band distribution of bacterial population in individual samples ranged from 20 to 26 (mean 22.40 ± 1.71 SD) in non-tumor where as 15 to 26 bands (mean 20.60 ± 3.10 SD) in tumor groups. The Mann–Whitney *U* test to compare the Shannon-Weaver indexes of diversity (H’) in non-tumor and tumor samples showed no significant differences (*p > 0.05, two-tailed*) in oral microbiota between two sample groups. The inter- group similarities were found to be 40% to 80% by cluster analysis (Figure 2). Most of the clinically distinct samples (non-tumor and tumor) from the same patients clustered together with exception of one sample (184_N and 184_T) as seen in their intensity profiles.

Similarity index (SI) was calculated based on the total number of high and low intensity bands per lane and position of band migration reflecting number of bands the two lanes have in common. The values signify similarities in bacterial composition between non-tumor and tumor groups (Table 1). The tumor samples (intra- group), 1457_T and 527_T showed total dissimilarity in their profiles despite sharing the same group. The band similarity correlation was highest in non-tumor and tumor tissue samples (inter- group), 142_N/142_T (77.27%) and 146_N/146_T (71.43%) from the same patient indicating that most of the microbiota were common at both the sites but there were changes in the bacterial composition. Chi-square test indicated significant differences in intra- and inter- groups bacterial profiles (*X*^2^ = 10.76, *p* = 0.005).

**Table 1:** Similarity index of DGGE fingerprinting pattern from two clinically distinct non-tumor and tumor groups

	**Similarity Index (%)**																			
	**140_N**	**140_T**	**64_N**	**64_T**	**1293_N**	**1293_T**	**211_N**	**211_T**	**184_T**	**527_N**	**527_T**	**146_N**	**146_T**	**184_N**	**164_N**	**164_T**	**142_N**	**142_T**	**1457_N**	**1457_T**
**140_N**	100																			
**140_T**	47.83	100																		
**64_N**	35.56	35.56	100																	
**64_T**	39.13	43.48	66.67	100																
**1293_N**	41.87	27.91	42.86	41.87	100															
**1293_T**	30	30	35.9	40	59.46	100														
**211_N**	31.11	31.11	36.37	44.45	38.1	30.77	100													
**211_T**	50	36.37	32.56	54.55	34.15	31.58	65.12	100												
**184_T**	41.87	27.91	33.33	37.21	50	32.43	42.86	58.54	100											
**527_N**	36.37	45.46	46.51	50	39.03	36.85	41.87	42.86	39.03	100										
**527_T**	42.11	31.58	32.43	42.11	34.29	31.25	43.25	44.45	45.72	50	100									
**146_N**	27.27	54.55	37.21	50	34.15	21.05	32.56	47.62	48.78	52.39	44.45	100								
**146_T**	36.37	54.55	37.21	54.55	34.15	26.32	55.81	57.15	48.78	42.86	50	71.43	100							
**184_N**	31.11	35.56	27.27	40	28.57	20.51	45.46	51.17	47.62	51.17	32.43	65.12	65.12	100						
**164_N**	20.41	36.74	29.17	28.57	26.09	37.21	25	25.53	26.09	12.77	19.51	38.3	12.77	33.33	100					
**164_T**	24.49	28.57	20.83	24.49	21.74	27.91	16.67	21.28	21.74	17.03	24.39	21.28	25.53	16.67	38.47	100				
**142_N**	34.05	34.05	30.44	25.53	31.82	43.91	17.39	35.56	40.91	13.33	30.77	40	35.56	30.44	56.01	36.01	100			
**142_T**	32.56	46.51	33.33	32.56	40	27.03	33.33	43.91	40	24.39	51.43	68.29	53.66	47.62	26.09	34.79	77.27	100		
**1457_N**	43.48	21.74	22.23	21.74	41.87	30	22.23	36.37	41.87	18.19	31.58	31.82	22.73	31.11	36.74	40.82	46.81	41.87	100	
**1457_T**	13.95	18.61	23.81	18.61	15	27.03	14.29	14.64	20	9.76	0	19.51	19.51	14.29	30.44	26.09	36.37	15	65.12	100

N–Non-tumor; T–Tumor.

The alterations in DGGE fingerprinting profiles indicated that different bacteria colonize the two oral sites, non-tumor and tumor of OSCC patients. This prompted us to conduct cloning and sequencing studies using 16S rDNA amplification to identify microbiotal populations at these sites. The clonal libraries with clinical distinctions were constructed with approximately 1200 high quality sequences from the rDNA inserts of non-tumor and tumor tissues. About 276 (~22.9%) sequences with <350 bases and 14 chimeric sequences (1.2%) were eliminated from analysis. The filtered 914 (75.9%) sequences of 350–900 bases from combined (non-tumor and tumor) library were characterized, of which 107 sequences (8.9%) with <98% sequence identity accounted for genus level classification and were uncharacterized at species level. The remaining 807 (67%) sequences having >98% sequence identity to 16S rRNA reference sequences in HOMD were classified to species level.

**Figure 3:**
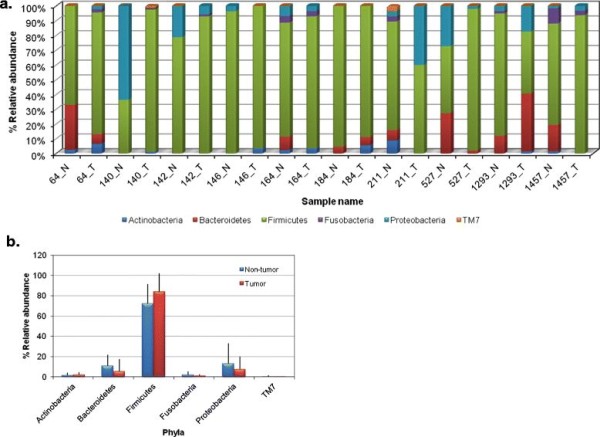
**Distribution of relative abundance of phyla in (a) Individual sample set, non-tumor and tumor sites of each OSCC patient and; (b) Cumulative non-tumor and tumor libraries, as detected by HOMD and RDP.** N–Non-tumor; T–Tumor.

**Figure 4:**
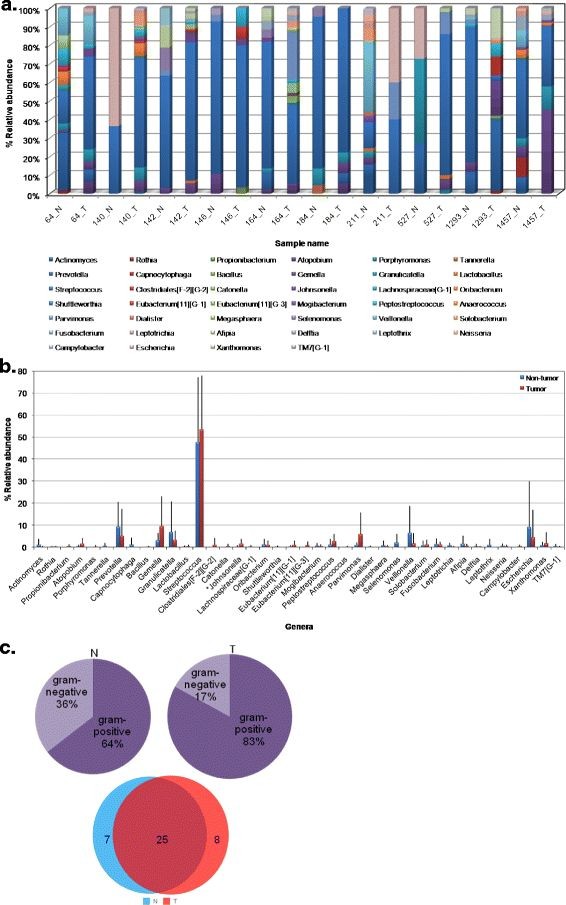
Distribution of relative abundance of genera at (a) Non-tumor and tumor sites of each OSCC subject; and (b) Cumulative non-tumor and tumor libraries, as detected by HOMD and RDP; (c) Pie-chart shows the relative prevalence of gram-negative and gram-positive bacteria and venn diagram depicts the genera in tissue samples of OSCC subjects.*p < 0.1. N–Non-tumor; T–Tumor. Pie-chart shows the relative shift of gram-negative and gram-positive microbiota in non-tumor and tumor tissue samples. Values in the venn diagram represent the genera shared by and exclusive to non-tumor and tumor tissue libraries.

Figure 3a shows the % distribution of phyla at tumor and non-tumor sites of the patient population. The filtered 914 sequences matched 6 bacterial phyla, in their degree of dominance, *Firmicutes*, *Bacteroidetes*, *Proteobacteria*, *Fusobacteria*, *Actinobacteria* and uncultivated *TM7* were detected in both non-tumor and tumor libraries (Figure 3b). The frequency of phylum *Firmicutes* was major in tumor tissues (85%) as compared to non-tumor tissues (74.6%) whereas the frequency of other phyla was higher in non-tumor library. The composition of bacterial communities at tumor site was different in comparison to the non-tumor site in most of the patients (Figure 4a). In combined library, 12 classes, 16 order, 26 families and 40 genera were observed and their relative distribution in individual non-tumor and tumor library is demonstrated in (see Additional file 1: Figure S1, Additional file 2: Figure S2, Additional file 3: Figure S3) and Figure 4b respectively. The most prevalent classes were *Bacilli* (66.6%) that includes order, *Lactobacillales* (54.8%) and *Bacillales* (11.8%) in tumor library while *Clostridia* (20.5%) and *Bacteroides* (11.8%) in non-tumor library.

The distribution of relative abundance of 40 representative genera in combined library (Figure 4b) was predominated by *Streptococcus* (50.8%), *Gemella* (11.6%), *Parvimonas* (4.6%), *Peptostreptococcus* (2.8%), *Xanthomonas* (2.4%), *Johnsonella* (1.6%), *Solobacterium* (1.6%), *Atopobium* (1.2%) and *Eubacterium[*[11]*][G-1]* (0.8%), in tumor library while *Prevotella* (11.6%), *Veillonella* (9.9%), *Granulicatella* (3.9%), *Escherichia coli* (2.4%), Oribacterium (2.2%), *Fusobacterium* (1.9%), *Actinomyces* (1.4%), *Megasphaera* (1.4%), *Afipia* (1.2%) and *Leptotrichia* (1.0%) in non-tumor library. Among others, genera *Capnocytophaga, Selenomonas* and *Leptothrix* were exclusive to non-tumor (control) tissues and *Eubacterium[*[11]*][G-3]*, *Campylobacter* and *Catonella,* confined only to tumor tissues. Figure 4c shows the relative shift from gram-negative to gram-positive microbiota by an increase of 19% in tumor tissue samples than in control non-tumor samples. Also, it was observed that the two groups shared 25 genera, while 7 genera were exclusive to non-tumor group and 8 genera to tumor group (Figure 4c).

**Figure 5:**
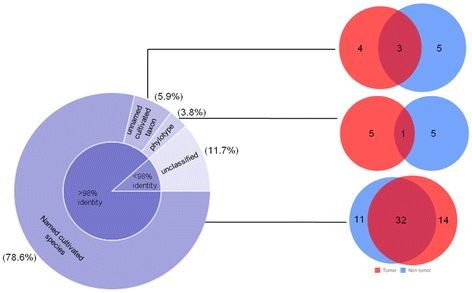
**Relative distribution of total bacteria (cultivable species and uncultured phylotypes) in tissues from non-tumor and tumor sites of OSCC subjects characterized by HOMD.** Core of pie chart shows percentage distribution of total 914 filtered sequences in terms of their % homology to curated 16S rRNA sequences in HOMD. Outer concentric of pie chart depicts the oral bacterial taxa in combined library; sequences with >98% identity: named cultured species (78.6%), unnamed cultured species (5.9%) and yet-uncultured phylotypes (3.8%); and sequences with <98% identity (11.7%) were considered as unclassified sequences characterized only to genus level. Venn diagrams correlates with the corresponding section of pie chart as indicated by line except for the unclassified sequences. Inset values in two subsets (non-tumor and tumor) represents observed bacterial species unique to that particular library. Values in overlapping section of subsets reflect oral taxa common to both sites.

The core of pie chart shows % distribution of 914 total sequences in terms of % homology to curated 16S rRNA sequences in HOMD (Figure 5). The outer concentric of pie chart depicts total oral bacterial taxa with >98% identity contributing to named cultivable species (78.6%), unnamed cultivable species (5.9%) and non-cultivable or uncultured phylotypes (3.8%) and the sequences with <98% identity are unclassified species (11.7%) characterized only to genus level. These total sequences in RDP showed homology with ~60% of uncultured phylotypes. Therefore, the sequences analyzed with HOMD were taken into consideration for species level identification. The venn diagrams (Figure 5) are embedded to corresponding section of pie chart except for the unclassified sequences and the inset values in two subsets (non-tumor and tumor) correlates to observed bacterial species unique to that particular library. The number of species shared or common to both the groups is seen in overlapping section of subsets.

**Figure 6:**
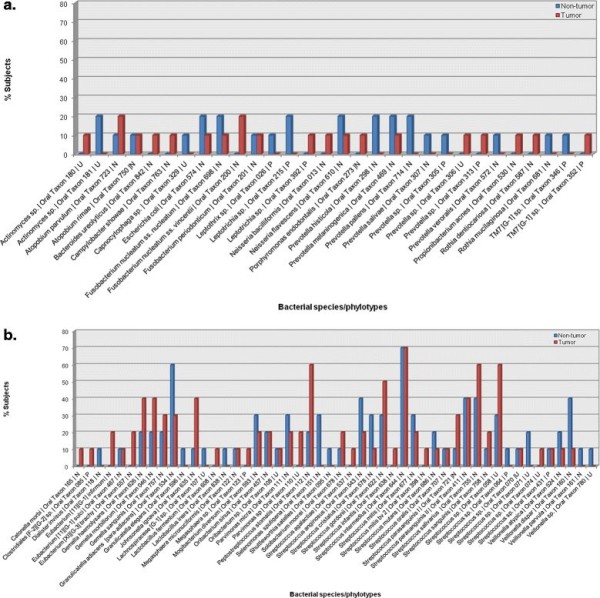
**Prevalence of bacterial species/phylotypes associated with non-tumor and tumor sites of OSCC subjects corresponding to phyla: (a) *Bacteroidetes*, *Proteobacteria*, *Fusobacteria*, *Actinobacteria*, uncultured *TM7*; and (b) *Firmicutes*, as detected by HOMD.**

In total, 80 bacterial species/phylotypes were detected, 57 in non-tumor and 59 in tumor library. The unnamed cultivable biota, *Actinomyces sp*. *oral taxon 181*, phylotype *Leptotrichia sp*. *oral taxon 215*, and certain named bacterial species, *Prevotella histicola*, *Prevotella melaninogenica*, *Prevotella pallens*, *Fusobacterium nucleatum ss. nucleatum*, *Escherichia coli* and *Neisseria flavescens* were detected at non-tumor site while *Atopobium parvulum* and *Fusobacterium nucleatum ss. vincentii* at tumor site (Figure 6a). The microbiota associated with phylum *Firmicutes* showed interesting switch in profile (Figure 6b). Species, *Granulicatella adiacens*, *Mogibacterium diversum*, *Parvimonas micra*, *Streptococcus anginosus*, *Streptococcus cristatus*, *Streptococcus mitis* and *Veillonella dispar* were prevalent at non-tumor site of the OSCC patients. The unnamed cultivable taxon, *Streptococcus sp*. oral taxon *058*, and named cultivable bacterial species, *Gemella haemolysans*, *Gemella morbillorum*, *Gemella sanguinis*, *Johnsonella ignava*, *Peptostreptococcus stomatis*, *Streptococcus gordonii*, *Streptococcus parasanguinis I*, *Streptococcus salivarius* were highly associated to tumor site. *Streptococcus sp*. oral taxon *071* and *Selenomonas sputigena* were confined to non-tumor site whereas *Parvimonas sp*. oral taxon *110*, *Eubacterium [*[11]*][G-1] infirmum* and *Eubacterium [XI][G-3] brachy* were exclusive to tumor site. *Streptococcus intermedius* was the most prevalent species. *Streptococcus parasanguinis II* and *Oribacterium sinus* were detected at both sites. Some observed bacterial species/phyloypes were less frequent in OSCC patients.

The species richness, coverage, diversity and evenness were estimated for two independent and combined set of libraries (Table 2). Shannon-Weaver and Simpson diversity indices revealed higher values indicating a huge species diversity in two libraries but no significant differences, Shannon diversity *t* test, *p* = 0.07 (*p* > 0.05). However, the richness estimators, Chao1 and ACE were higher in tumor library than in non-tumor library. Evenness was greater with non-tumor samples as compared to tumor samples suggesting less abundant species at tumor site. Good’s coverage of the combined library was ~98% suggesting that 2 additional phylotypes would be recognized if 100 more clones were screened. Individual-based rarefaction curves calculated using PAST for the two library sets showed asymptote curve (see Additional file 4: Figure S4a) at actual community richness depicting that libraries were large enough to represent majority of oral bacterial species in the sampled subsets. Rank abundance curves were plotted to compare how well the communities have been sampled (see Additional file 4: Figure S4b). A long right-hand tail indicated rare species with few abundant species in both libraries.

**Table 2:** Richness, diversity indices and coverage estimation in individual and combined libraries

	**N**	**T**	**Combined**
	**(*n = 10*)**	**(*n = 10*)**	**(*n = 20*)**
No. of clones	414	500	914
Species/phylotypes (S)	57	59	80
Singletons	16	22	21
Doubletons	9	7	13
Chao1 estimator of species richness	71.22	93.57	96.96
Chao1 standard deviation	9.34	20.56	9.69
ACE estimator of species richness	68.59	83.76	97.78
Shannon’s index for diversity (H)	3.37	3.20	3.47
Simpson’s index for diversity (1-D)	0.94	0.92	0.94
Evenness (e^H/S)	0.51	0.42	0.40
Good’s estimator of coverage (%)	96.14	95.6	97.7

N–non-tumor; T–tumor; Combined–non-tumor and tumor; n–number of samples.

## Discussion

Bacteria have the capacity to penetrate and invade various epithelial cells colonizing and inducing inflammation which may plausibly associate to cancer progression [63,64]. For example, *H. pyroli* have been known to be associated to inflammation of gastric mucosa leading to gastritis, peptic ulcers, gastric carcinoma and gastric mucosa-associated lymphoid tissue (MALT) lymphomas [18]. Recently, a link between periodontal disease and cancer has been suggested and the hypothetical proposition is the chronic nature of inflammatory process underlying periodontitis [64,65]. To the best of our knowledge, this is the first report using DGGE in parallel to sequencing for profiling bacterial flora and compares the diversity in non-tumor and tumor tissues from same individual. Here, we used homogenous population to control various confounding factors and hence did not compare bacterial colonization within healthy individuals but screened the normal mucosa collected from the same subject. Thus the role of microbes in oral diseases can be predicted looking at the changes in indigenous (non-tumor) and diseased (tumor) microenvironments. DGGE allows rapid assessment of bacterial diversity in various environments and we have extensively used this technique in our earlier studies on saliva and cariogenicity [45,51,52]. The fingerprints represents separation of DNA fragments of same length based on differences in nucleotide and each individual band relates to one or more bacterial species [66].

In this study, the observed differences in DGGE profiles of inter- group, 22.73%–90.24% among non-tumor and tumor groups, and intra- group diversity, 34.88%–87.23% within non-tumor group and 41.46%–100% within tumor group, signified some underlying changes in bacterial colonization of the tissues. Thus, even slight differences in bacterial profile of non-tumor and tumor tissues seem significant as samples were procured from the same individual. It is not surprising that fingerprints showed no significant differences in mean total number of bands. DGGE is a semi-quantitative method and the band intensities are also influenced by 16S rRNA gene copy numbers or co-migration of two or more sequence types or combination of these [67,68]. However, the relative distribution of more intense bands may represent species indigenous and abundant in oral microenvironment. The less intense bands indicated indigenous but less rich species or species in low numbers. Some species that were found to be higher in one group were either less abundant or even absent in other group. This indicates close interactions within the microbial communities’ along with relative microbial shift at two target sites. Our earlier study on DGGE fingerprints of saliva samples from OSCC and healthy subjects have shown significant group-specific clusters despite inter- subject variability that may enable to differentiate OSCC from healthy subjects [40].

This was further substantiated by the results of 16S clonal analysis showing relatively distinct bacterial affiliations at non-tumor and tumor sites of OSCC subjects. *Firmicutes* were highly prevalent at tumor site as observed earlier [37,38,40]. About 25 genera were common to both sites. There were differences in the relative abundance of bacteria, however, no statistically significant differences in phylogenies were detected at tumor and non-tumor sites of the OSCC patients except for genus *Johnsonella* (*p < 0.1*). The bacterial species associated with tumor tissues were far more diverse than that previously shown by culture-dependent [10,33-36] and culture-independent studies [38]. The predominance of gram-positive bacteria relative to gram-negative bacteria suggests differences in the bacterial communities at two clinically distinctive sites. These oral bacteria may act as a primary trigger or precursor of mucosal lesions or secondary invaders in non-infectious mucosal lesions [33].

An interesting observation related to clonal analysis was that the sequences when matched with the two known databases, RDP and HOMD for highest similarity showed similar results up to genus level. But at species level, the uncultivable phylotypes detected were 3.83% and ~60% by HOMD and RDP respectively. This may be due to differences in basic structure of two databases. Unlike RDP, HOMD is a curated database with 626 species and phylotypes based on 98.5% similarity cutoffs of full 1540-base 16S rRNA sequences and each oral taxon assigned a specific number.

Most of the cultivable bacteria, *Actinomyces sp*. oral taxon *181*, *Streptococcus sp*. oral taxon *071*, *P. histicola*, *P. pallens*, *Selenomonas sputigena*, *V. dispar* and phylotype, *Leptotrichia sp*. oral taxon *215* present in non-tumor tissues are known putative representatives of predominant genera in healthy oral microbiome [69]. *Prevotella* has earlier been associated with different types of endodontic infections [70] and *Leptotrichia* an opportunistic pathogen with bacteremia or sepsis producing lactic acid as a major metabolic end product [71]. *Granulicatella adiacens* which was highly prevalent in non-tumor group is also a known agent of endocarditis [72]. *S. intermedius* was predominant in 70% of OSCC subjects at both non-tumor and tumor sites. *S. parasangunis II* and *O. sinus* were also present at both sites. *Oribacterium species* are weakly fermentative forming metabolic end products, acetic and lactic acid [73]. *S. anginosus* detected at 4 non-tumor and 2 tumor sites has been reported earlier in OSCC specimens [36,38] and saliva of alcoholics [74]. The *Streptococcus anginosus* group comprised of three species, *S. anginosus*, *S. constellatus* and *S. intermedius* and are normal flora in humans, these bacteria are pathogens associated strongly with abscess formation and with infection in multiple body sites [75]. Assacharolytic *Eubacterium* and closely related strains found in our study at tumor sites are major bacterial groups in oral lesions and play important role in infections of root canal and periodontal pockets and use proteins and peptides derived from tissues and blood as energy source [76]. Also, *Atopobium*, *F. nucleatum ss. vincentii* and *Parvimonas* have been associated with endodontic infections or periodontitis [40,77,78]. Together, these observed species at tumor sites substantiates its association with some early primary infection of lesion that may act as a trigger for tumor initiation and progression [79].

*Streptococci* were more prevalent at tumor sites as also reported earlier [10,34,35,80]. We observed *Streptococcus sp*. oral taxon *058*, *Peptosteptococcus stomatis*, *S. salivarius*, *S. gordonii*, *G. haemolysans, G. morbillorum*, *J. ignava* and *S. parasanguinis I*, to be associated with tumor site. Van Houte et al. [81,82] identified significant populations of *Streptococci* which produced large amounts of acid (pH < 4.2 in broth) in both coronal caries and root-surface caries. *Streptococci* are saccharolytic producing short chain organic acid from carbohydrates, thus lowering the pH of their local environment [83] and also aciduric *P. stomatis* found in oral cavity is weakly saccharolytic and produces fermented products, acetic, butyric, isobutyric, isovaleric and isocaproic acids [84]. These microbiota may contribute to the acidic and hypoxic microenvironment of tumors [85,86] and promote bacterial colonization. Anaerobes, *Gemella* species like any other commensal are opportunistic pathogens known to cause serious local and systemic infections mainly in immune-suppressed patients [40,87] were detected at tumor sites [35,40]. *J. ignava* can be a predicted new pathogen not detected in earlier studies and known to be associated with gingivitis and periodontitis [88].

Studies have shown association of tooth loss or periodontal diseases and oral cancer [89-91]. Periodontal disease is often linked to cardiovascular disease, low-birth weight complications in pregnancy, diabetes and pulmonary disease and certain cancers including oral cancer [79]. The common factor between periodontal disease and cancer is inflammation driven by bacteria. At this point of time, it is not clear whether changes in bacterial colonization act as a trigger to lesion formation. However, once the lesion is formed which may be spontaneous or due to underlying changes in the host tissues as a result of external factors such as smoking, drinking or oral health, specific oral bacteria can colonize and induce inflammation. Oral bacteria have shown ability to adhere, co-aggregate or colonize on specific surfaces in oral cavity representing tissue tropism as reported in several studies [92,93].

The involvement of infection-triggered inflammations has been estimated in the pathogenesis of approximately 15–20% of human tumors [17,94]. Recently, it has been shown that two specific bacterial subpopulations, *Enterobacteriaceae* and *Tenericutes* lead to increase in methylation of multidrug resistance gene1 (MDR1 gene) and bacterial-triggered inflammation that correlates with regional nodal metastases over adjacent normal mucosa [63].

Mager et al. [93] demonstrated significant differences in the bacterial profiles of 40 oral cultivable species on soft and hard tissues in healthy subjects and found distinct profiles of the soft tissues than those of supragingival and subgingival plaques. Using culture-independent molecular technique, Aas et al. [92] analyzed samples of nine sites from five healthy subjects and showed the site and subject specificity of bacterial colonization in the healthy human oral cavity. In our study too, despite the homogenous population, several species were site-specific, while others were subject-specific and undergo succession from health to disease. Hence, even a slight distinction in bacterial community at non-tumor and tumor sites has significance as the samples were from two adjoining sites of same OSCC subject. The underlying species-specific shift implicates alterations in bacterial colonization at tumor sites. The translocation of bacteria from oral cavity to cervical lymph nodes and more in metastatic than in uninvolved nodes in oral cancer patients has been reported by Sakamoto et al. [35].

## Conclusions

Together, the results indicate that certain bacterial species/phylotypes detected in this study may play a role in triggering chronic inflammation in oral cavity and possibly be associated at different stages of cancer [95]. This may be due to disrupted oral mucosal surface allowing bacterial invasion and perhaps serve as point of entry to the regional lymph nodes [33,35]. This indicates that though the bacterial biota were commensals of oral cavity and may become pathogenic when their balance is disturbed. Microbial shift or dysbiosis has been implicated in some diseases due to unequal ratio of beneficial symbionts to pathogens [96]. This study recognized association of some new bacterial species, like *J. ignava* not detected earlier in tumor samples by culture- dependent or independent methods. However, these studies were performed with limited sample size. Therefore, further investigation with larger sample size using high throughput sequencing would validate these findings and broaden our perspective on bacterial association and oral cancer.

## Competing interests

The authors declare that they have no competing interests.

## Authors’ contributions

SP participated in the design, implementation, analysis, interpretation of the results and writing the manuscript. XJ participated in implementation and analysis. YL participated in analysis of DGGE profiles. CE, RY and BS participated in collecting and providing the samples. XL participated in interpretation of the results and writing the manuscript. DS conceived of the study and participated in the design, implementation, analysis, interpretation of the results and writing the manuscript. All authors read and approved the final manuscript.

## Supplementary Material

Additional file 1 **Figure S1.** Distribution of relative abundance of classes detected by HOMD and RDP in tissue samples from non-tumor and tumor sites of OSCC subjects. Click here for file

Additional file 2 **Figure S2.** Distribution of relative abundance of order detected by HOMD and RDP in tissue samples from non-tumor and tumor sites of OSCC subjects. Click here for file

Additional file 3 **Figure S3.** Distribution of relative abundance of families detected by HOMD and RDP in tissue samples from non-tumor and tumor sites of OSCC subjects. Click here for file

Additional file 4 **Figure S4.** (a) Individual-based rarefaction; and (b) Rank abundance curves for bacterial species associated with non-tumor tissue and tumor tissue libraries. Click here for file
