# Supplementary material for: Comparison of oral microbiota in tumor and non-tumor tissues of patients with oral squamous cell carcinoma
Source: BMC Microbiol. 2012 Jul 20;12:144. doi: 10.1186/1471-2180-12-144 (PMC3507910; doi:10.1186/1471-2180-12-144)
Supplement: Additional file 3 — Figure S3. Distribution of relative abundance of families detected by HOMD and RDP in tissue samples from non-tumor and tumor sites of OSCC subjects. [file 1471-2180-12-144-S3.doc]

**Additional file 3: Figure S3. Distribution of relative abundance of families detected by HOMD and RDP in tissue samples from non-tumor and tumor sites of OSCC subjects.**

**
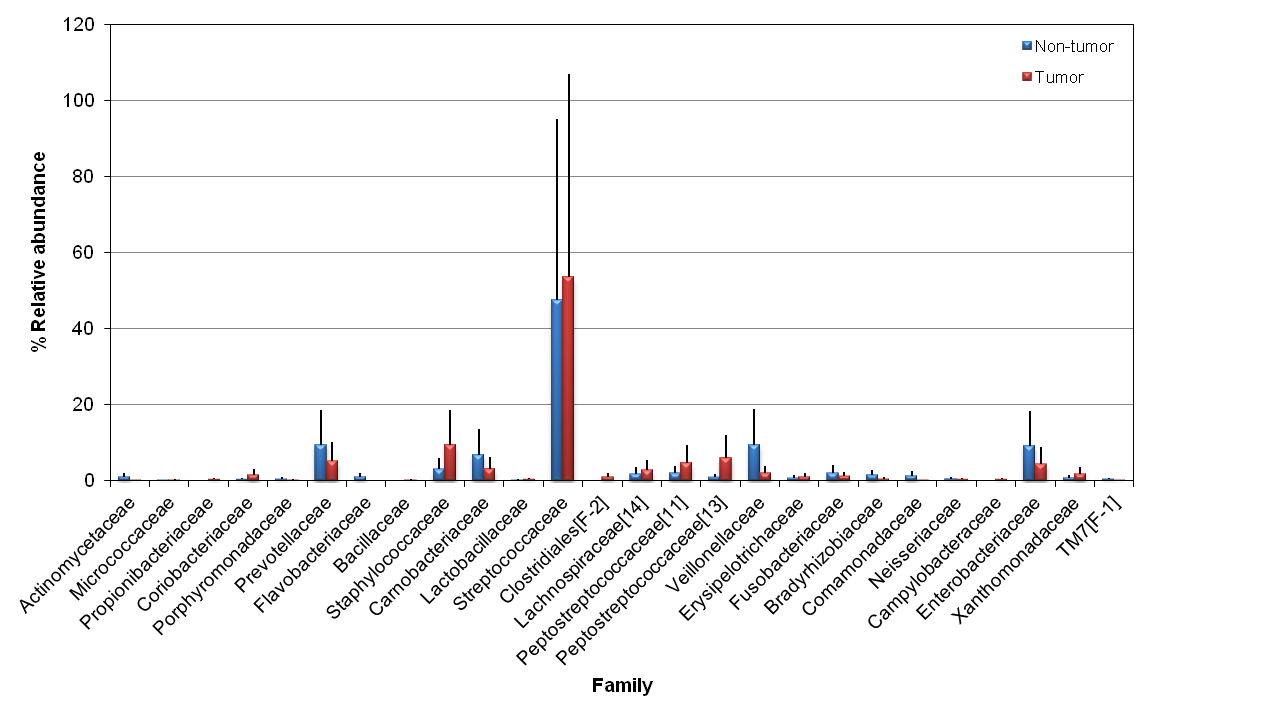
**
