# Supplementary material for: Comparison of oral microbiota in tumor and non-tumor tissues of patients with oral squamous cell carcinoma
Source: BMC Microbiol. 2012 Jul 20;12:144. doi: 10.1186/1471-2180-12-144 (PMC3507910; doi:10.1186/1471-2180-12-144)
Supplement: Additional file 4 — Figure S4. (a) Individual-based rarefaction; and (b) Rank abundance curves for bacterial species associated with non-tumor tissue and tumor tissue libraries. [file 1471-2180-12-144-S4.doc]

**Additional file 4: Figure S4. (a) Individual-based rarefaction; and (b) Rank abundance curves for bacterial species associated with non-tumor tissue and tumor tissue libraries.**


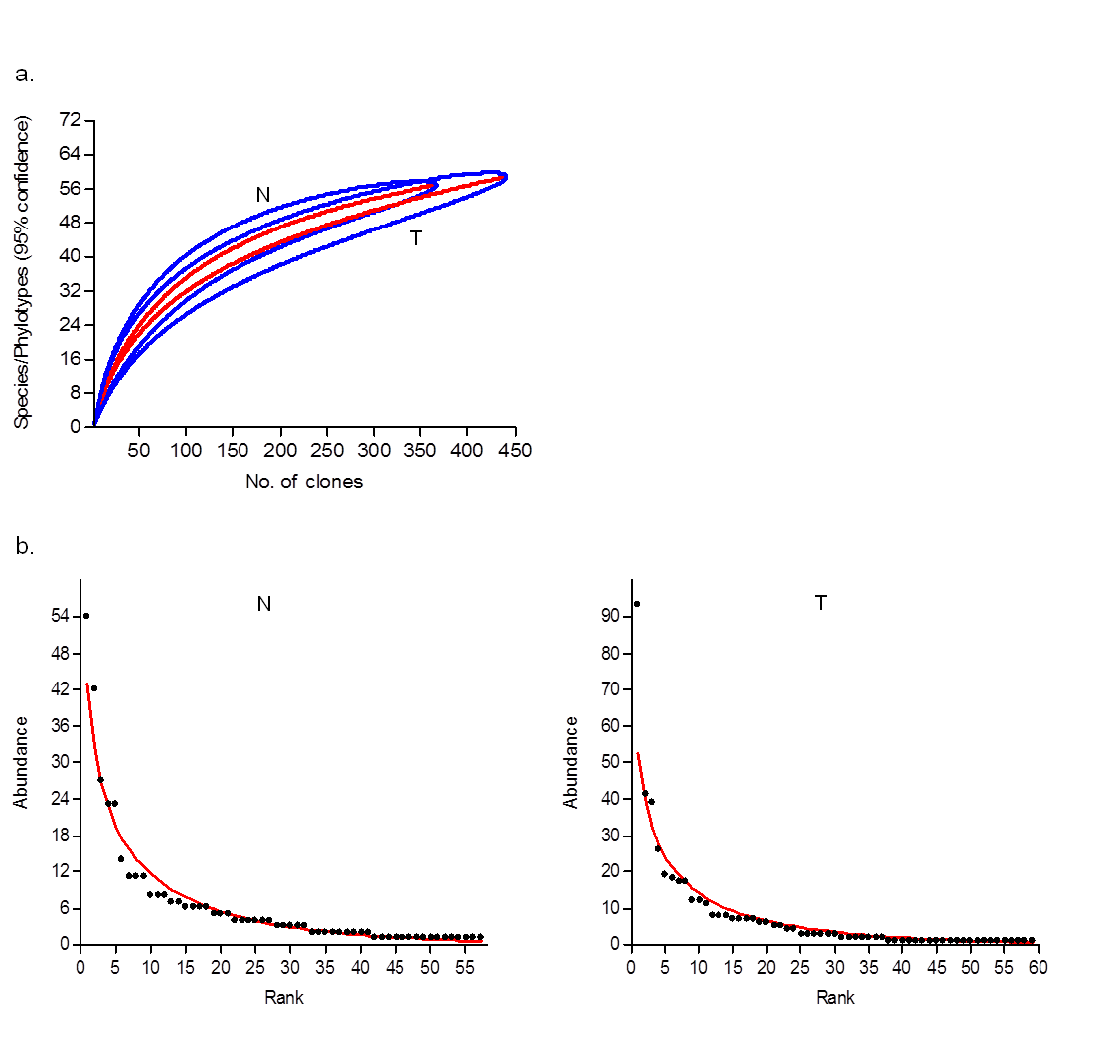
N – Non-tumor; T – Tumor
